# Emotional regulation and mental health profiles among Chinese junior middle school students: evidence for the dual-factor model

**DOI:** 10.3389/fpsyg.2025.1708381

**Published:** 2026-01-07

**Authors:** Jichang Guo, Yanpei Pan, Yan Zhao, Jiaoyang Liu, Yiduo Ye

**Affiliations:** 1 School of Education Science, Minzu Normal University of Xingyi, Xingyi, China; 2 School of Literature and Journalism, Minzu Normal University of Xingyi, Xingyi, China; 3 School of Psychology, Fujian Normal University, Fuzhou, China

**Keywords:** junior middle school students, dual-factor model of mental health, emotional regulation, 3-step multinomial logistic regression, latent profile analysis

## Abstract

**Objective:**

This study explored latent mental health profiles among adolescents in southwestern China and the association with emotional regulation using the dual-factor model framework.

**Methods:**

1,682 junior middle school students completed the *Emotion Regulation Questionnaire* (*ERQ*), *Center for Epidemiological Studies Depression Scale* (*CES-D*), and *Satisfaction With Life Scale* (*SWLS*). Latent Profile Analysis (LPA) identified mental health profiles, and 3-step multinomial logistic regression examined the relationship between emotion regulation and the profiles recognized by LPA.

**Results:**

LPA revealed three profiles: Troubled (31.51%, high negative symptoms/low well-being), complete mental health (61.30%, low negative symptoms/high well-being), and more troubled (7.19%, severe negative symptoms/extremely low well-being). Cognitive reappraisal positively predicted complete mental health (vs. Troubled; *OR* = 1.096, *p* < 0.001) and negatively predicted more troubled (*OR* = 0.899, *p* < 0.001). Expressive suppression negatively predicted complete mental health (*OR* = 0.863, *p* < 0.001) and positively predicted more troubled (*OR* = 1.092, *p* < 0.001).

**Conclusion:**

Three distinct profiles emerged, differing from the traditional dual-factor model. Cognitive reappraisal protects mental health, while expressive suppression correlates with poorer outcomes, highlighting the need for targeted interventions promoting cognitive reappraisal.

## 1 Introduction

Adolescent mental health plays a crucial role in their overall development, influencing academic achievement, social integration, and long-term psychological resilience. Mentally healthy adolescents demonstrate better academic performance, prosocial behaviors (Suldo and Shaffer, 2008; Antaramian et al., 2010). Research consistently highlights emotional regulation as a critical predictor of mental health outcomes (Dryman and Heimberg, 2018; Elhai et al., 2018; Vally and Ahmed, 2020; Liang et al., 2022; Liu et al., 2024; Sadeghian et al., 2024; Guo and Pan, 2025). As a core psychological mechanism, emotional regulation shapes responses to positive and negative emotions, affecting mental health conditions’ onset, severity, and persistence.

### 1.1 Emotional regulation and adolescents’ mental health

Emotional regulation refers to the extrinsic and intrinsic processes responsible for monitoring, evaluating, and modifying emotional reactions to accomplish one’s goals, encompassing a broad range of regulatory processes including the regulation of emotions by oneself versus others and the regulation of the emotion itself versus its underlying features (Thompson, 1994; Thompson and Calkins, 1996).

Emotion regulation strategies are processes through which individuals modulate their emotions to achieve goals, with a key classification distinguishing adaptive from maladaptive approaches. Adaptive strategies like cognitive reappraisal involve adjusting one’s thinking about situations to mitigate emotional impact (Gross, 1998) and significantly benefit mental health by modulating emotional responses and adaptive functioning. Research demonstrates it is positively associated with psychological well-being, serving as a protective factor against depression and anxiety (Garnefski et al., 2001; Martin and Dahlen, 2005; Aldao et al., 2010). Frequent users experience more positive emotions, employ effective coping mechanisms, report fewer depressive symptoms and greater life satisfaction (Joormann and Tanovic, 2015), and adolescents adept at this strategy show lower depressive symptoms and higher life satisfaction (Garnefski and Kraaij, 2006). For instance, it predicts reduced depression by fostering school connectedness (Zhao and Zhao, 2015).

In contrast, maladaptive strategies such as expressive suppression entail inhibiting emotional expression (Gross, 2002; Gross and John, 2003) and have a complex, culturally contingent association with mental health. Some studies link it to increased negative affect, physiological arousal, cognitive load, and elevated depression/anxiety risk (Gross and John, 2003; Aldao et al., 2010), with reliance correlated to more internalizing problems in adolescents (Gross and John, 2003; Garnefski and Kraaij, 2006). However, negative outcomes are not universal (Balzarotti et al., 2010; Cabello et al., 2013), as patterns differ across collectivist and individualist societies (Potthoff et al., 2016; Zhou et al., 2020; Simon et al., 2024; Zhao and Hu, 2024). For example, cultural acceptance of emotional restraint may mitigate its adverse effects in Eastern cultures like Japan (Soto et al., 2016). Overall, emotional regulation is critical for mental health. Poor regulation amplifies stress reactivity and elevates anxiety and depression risks (Ahmed et al., 2015), whereas developing effective strategies fosters wellness and guards against mental disorders.

### 1.2 Dual-factor model of mental health

Introduced by Greenspoon and Saklofske (2001), the dual-factor model (DFM) of mental health conceptualizes mental health by integrating subjective well-being and psychopathology as distinct yet complementary dimensions, with two core indicators. Positive indicators correspond to subjective well-being (e.g., life satisfaction, positive affect; Greenspoon and Saklofske, 2001; Keyes, 2002; Suldo and Shaffer, 2008) and negative indicators refer to psychopathology, including internalizing problems (e.g., depression, anxiety) and externalizing problems (e.g., aggression, rule-breaking; Suldo and Shaffer, 2008).

The model classifies individuals into four mental health groups according to the cutoff scores of positive and negative indicators: (1) complete mental health (high well-being, low psychopathology), (2) vulnerable (low well-being, low psychopathology), (3) symptomatic but content (high well-being, high psychopathology), and (4) troubled (low well-being, high psychopathology). These groups demonstrate distinct outcomes in academic performance, social functioning, and resilience, underscoring the importance of assessing both dimensions simultaneously (Suldo and Shaffer, 2008; Lyons et al., 2012).

Over the past two decades, this framework has gained robust empirical support across children, adolescents, and adults (Greenspoon and Saklofske, 2001; Antaramian et al., 2010; Eklund et al., 2010), with consistent identification of four prototypical subgroups (Suldo and Shaffer, 2008; Antaramian et al., 2010). It has also been validated in Chinese youth (Xiong et al., 2017; Zhou et al., 2020), as mental health profiles are linked to academic self-efficacy and emotional regulation. Systematic reviews (Magalhães, 2024) confirm 85% of dual-factor studies validate the four-group structure, with complete mental health as the most prevalent category, supporting integrated assessment protocols that emphasize both symptom reduction and well-being enhancement. Meanwhile, applicable in education, relevant interventions benefit adolescent well-being and reduce symptoms (Antaramian et al., 2010).

Despite its contributions, the model faces challenges. The reliance on cutoff scores to dichotomize continuous measures of psychopathology and well-being has been criticized for its methodological limitations. Magalhães (2024) noted that arbitrary cutoff thresholds may oversimplify the complexity of mental health constructs, potentially misclassifying individuals near the boundaries. Petersen et al. (2020) further emphasized that cutoff-based approaches lack sensitivity to nuanced variations, which could obscure transitions between mental health states in longitudinal analyses. Additionally, Moore et al. (2019) highlighted inconsistencies in cutoff criteria across studies, leading to variability in subgroup prevalence rates and reducing cross-study comparability.

While the DFM’s four-group structure is robustly supported across diverse adolescent samples (Greenspoon and Saklofske, 2001; Suldo et al., 2016), the use of cutoff scores remains a contentious issue, necessitating more nuanced methodologies such as latent profile analysis, to enhance precision and clinical utility.

### 1.3 Latent profile analysis for dual-factor model of mental health

Although numerous studies support the dual-factor model of mental health, Kim et al. (2018) contended that the dichotomous cutoff scores approach artificially imposes a four-group solution and fails to fully capture the complexity of adolescents’ mental health, prompting subsequent studies to investigate the model via data-driven, person-centered methods like latent class analysis (LCA) or latent profile analysis (LPA). LCA/LPA enable researchers to identify adolescent groups with distinct score patterns across indicators while offering greater flexibility in the number of identifiable groups (Muthén and Muthén, 2000; Nylund et al., 2007; Gregory et al., 2024), and they have emerged as critical methodological tools for identifying heterogeneous subgroups within this framework by transcending traditional unidimensional approaches to mental health assessment.

Empirical studies consistently identify 3–5 distinct mental health profiles, with variations linked to sample characteristics and methodology. Jiang et al. (2023) and Zhou et al. (2020) identified three subgroups (flourishing, vulnerable, troubled) among Chinese university students and early adolescents via LPA, with no symptomatic but content group. Clark and Malecki (2022) found three matching profiles (complete mental health, symptomatic but content, troubled) in US adolescents, omitting the vulnerable group. Other studies report 4–5 subgroups, for example, Kim et al. (2018) distinguished four subtypes among Korean primary students; Moore et al. (2019) identified four groups in US middle/high schoolers, with grade-varying proportions; Gregory et al. (2024) detected five profiles.

Typological differences reflect contextual factors. Early adolescent studies (e.g., Zhou et al., 2020) exclude the symptomatic but content group, which emerges in later adolescence (Moore et al., 2019; Clark and Malecki, 2022). Chinese samples feature a vulnerable group that is absent in US studies, while larger samples and broader indicators (e.g., Gregory et al., 2024) enable finer classifications. These context-bound variations highlight the model’s strength in capturing heterogeneity as well as its lack of a universal typology, necessitating systematic exploration to clarify profile manifestations and develop generalizable frameworks.

### 1.4 The present study

Currently, a significant amount of research has examined the latent classes of the DFM of mental health in various groups and the impact of emotional regulation on mental health. Additionally, studies have explored the distinct roles of cognitive reappraisal and expressive suppression in different cultural contexts. However, there is a lack of research on how cognitive reappraisal and expressive suppression influence the potential categories of mental health. Exploring these aspects in adolescents in southwestern China also holds significant importance.

Therefore, the objectives of this study are to explore: (a) the latent profiles of mental health and the prevalence of different mental health profiles in adolescents in southwestern China; (b) the relationship between demographic characteristics, emotional regulation and the latent profiles of mental health. Based on the past literature, we expect to identify similar types to DFM of mental health and meanwhile different demographic characteristics and emotional regulation will have different effects on the latent categories.

## 2 Methods

### 2.1 Participants

A total of 1800 questionnaires were initially distributed to junior middle school students in six public schools in Guizhou Province, China. Finally, 1763 questionnaires were collected, with a response rate of 97.94%. After excluding 81 invalid responses (e.g., patterned answering), data from 1,682 valid questionnaires submitted by participants (930 males, 724 females, 28 missing gender information) were retained for analysis. Participants completed three standardized questionnaires and a demographic survey during March 2025. Written informed consent was obtained from all participants and their legal guardians prior to data collection. The participants had an average age of 14.37 years (SD = 1.23), ranging from 12 to 18 years. Participation was voluntary and anonymous, and uncompensated.

### 2.2 Measures

#### 2.2.1 Demographic survey

The demographic survey includes the items such as gender, age, grade and family location.

#### 2.2.2 Emotion regulation questionnaire

The *ERQ*, developed by Gross and John (2003) and revised by Wang et al. (2007), includes 10 items across two core dimensions: Cognitive Reappraisal (CR) and Expressive Suppression (ES), with a total of 10 items. Six items (items 1, 3, 5, 7, 8, and 10) assess the frequency of individual use of cognitive reappraisal strategies, while four items (items 2, 4, 6, and 9) focus on the use of expressive suppression strategies. Each item is rated on a 7-point Likert-type scale ranging from 1 (completely disagree) to 7 (completely agree). Higher scores on the cognitive reappraisal dimension indicate a greater tendency to use cognitive reappraisal strategies to regulate emotions, while higher scores on the expressive suppression dimension indicate a higher frequency of using expressive suppression strategies. In this study, the Cronbach’s ɑ for the scale was 0.657, and the confirmatory factor analysis (CFA) shows that χ^2^/*df* = 5.946, *SRMR* = 0.037, *CFI* = 0.938, *TLI* = 0.918, *RMSEA* = 0.054, indicating good reliability and validity.

#### 2.2.3 Center for epidemiological studies depression scale

The *Center for Epidemiological Studies Depression Scale* (*CES-D*), developed by Radloff (1977), consists of 20 items, which were originally categorized into four symptom groups: Depressed Affect (DA), Somatic Complaints (SC), Interpersonal Problems (IP), and Positive Affect (PA). The *CES-D* can be interpreted in terms of three symptom dimensions among Chinese adolescents: Depressed affect (DA), Positive affect (PA), Somatic complaints (SC; Wang et al., 2013). So three-factor *CES-D* was utilized in this study. The respondents reported how frequently they experienced each of the symptoms during the past week using a 4-point scale with 1 (rarely or less than 1 day) to 4 (most or all of the time or 5 ~ 7 days). The total score is calculated by summing up all 20 items, ranging from 20 to 80 points, with higher scores indicating more severe depressive symptoms. The Cronbach’s ɑ of *CES-D* in this study was 0.863 and the CFA of three-factor model shows that χ^2^/*df* = 4.700, *SRMR* = 0.035, *CFI* = 0.929, *TLI* = 0.919, *RMSEA* = 0.047, indicating good reliability and validity.

#### 2.2.4 Satisfaction with life scale

The *Satisfaction With Life Scale* (*SWLS*), developed by Pavot and Diener, includes five sentences for participants to rate on a 7-point Likert-type scale ranging from 1(strongly disagree) to 7(strongly agree), with a total score range of 5 to 35 points, where higher scores indicate higher levels of life satisfaction (Diener et al., 1985; Pavot and Diener, 1993). In this study, the Cronbach’s ɑ of the scale was 0.856 and the CFA with a slight modification shows that χ^2^/*df* = 4.789, *SRMR* = 0.010, *CFI* = 0.996, *TLI* = 0.991, *RMSEA* = 0.048, indicating good reliability and validity.

### 2.3 Statistical analysis

Demographics, *ERQ*, *CES-D* and *SWLS* scores were summarized with descriptive statistics using SPSS 26.0. LPA was performed in M*plus* 8.3 to identify empirically-driven latent profiles of mental health among adolescents based on the three *CES-D* subscales and *SWLS* scores.

According to prior recommendations (Spurk et al., 2020; Kong and Zhang, 2023; Zheng and Zhang, 2024), the following criteria were considered to determine the number of latent profiles: Akaike Information Criteria (AIC), Bayesian Information Criterion (BIC), Adjusted Bayesian Information Criterion (aBIC), Lo–Mendell–Rubin likelihood ratio test (LMR), Bootstrapped likelihood ratio test (BLRT), entropy and smallest class size. Specifically, smaller AIC, BIC, and aBIC indicate better model fit; a significant LMRT and BLRT result (*p* < 0.05) indicates that the model with the k profiles fits better than the *k* - 1 one; entropy values, ranging from 0 to 1, represent the accuracy of classification, and it is generally considered that values >0.80 imply high accuracies. That the smallest class should involve at least 5% of the total sample should also be considered (Wendt et al., 2019).

We use a 3-step approach (Asparouhov and Muthén, 2014), which can take into account categorical error, to check the link between the predictors and mental health profiles. In other words, tests of categorical latent variable multinomial logistic regressions using the 3-step procedure, with the first class as reference group, were used to test the relationship between emotional regulation and mental health profiles identified in this study.

Besides, CFA was preformed to get fit indices of *ERQ*, *CES-D* and *SWLS* in M*plus* 8.3. The standardized values of the three *CES-D* subscales and *SWLS* scores were used to perform LPA to make the profiles to be explained clearly and easily.

## 3 Results

### 3.1 Common method bias test

The Harman test (Podsakoff et al., 2003) was used to test the common method bias probably existing in this study. Exploratory factor analysis were performed on *ERQ* (10 items), *CES-D* (20 items) and *SWLS* (5 items) datasets, and then we checked the results of unrotated factor analyses. The results showed that a total of 6 factors with eigenvalues >1 were extracted from 35 items, and the explained percentage of variance of the first factor was 24.24%, which was lower than the critical criterion of 40%, so the common method bias had no impact on the data in this study.

### 3.2 Characteristics of the participants

Table 1 shows the characteristics of the participants, including sample size and percentage of total, average score on all variables and its standard deviation.

**Table 1:** Characteristics of the participants.

Variables	Gender		Age						Grade			Location	
	Male	Female	12	13	14	15	16	≥17	Grade 1	Grade 2	Grade 3	Countryside	Urban
Sample size (%)	930 (55.29)	724 (43.04)	56 (3.33)	394 (23.42)	421 (25.03)	418 (24.85)	250 (14.86)	58 (3.45)	538 (31.99)	438 (26.04)	700 (41.62)	818 (48.63)	648 (38.53)
Cognitive reappraisal	28.08 (6.23)	26.45 (6.08)	25.34 (6.80)	26.78 (6.17)	27.86 (6.15)	27.58 (6.26)	27.71 (5.85)	26.02 (4.85)	27.04 (6.28)	27.75 (5.99)	27.38 (6.31)	27.56 (6.16)	27.12 (6.39)
Expressive suppression	13.84 (5.26)	13.61 (5.43)	13.63 (5.43)	14.04 (5.38)	12.57 (4.96)	14.03 (5.28)	14.19 (5.70)	14.07 (4.13)	14.60 (5.25)	12.32 (5.14)	13.95 (5.31)	13.44 (5.28)	14.10 (5.36)
Somatic complaints	16.26 (5.01)	18.16 (6.42)	19.02 (6.41)	17.85 (6.07)	15.96 (5.48)	16.70 (5.43)	17.39 (5.48)	17.91 (4.91)	18.44 (6.23)	15.32 (4.92)	17.19 (5.53)	16.49 (5.39)	17.74 (6.08)
Depressive affect	9.69 (3.14)	10.23 (3.43)	11.19 (3.62)	10.36 (3.31)	9.50 (3.27)	9.66 (3.23)	9.82 (3.04)	10.83 (3.35)	10.64 (3.41)	9.04 (3.01)	9.94 (3.22)	9.56 (3.07)	10.25 (3.33)
Positive affect	7.88 (2.48)	8.29 (2.52)	8.70 (2.58)	8.24 (2.53)	8.07 (2.60)	7.86 (2.46)	7.88 (2.33)	8.10 (2.39)	8.46 (2.51)	7.54 (2.43)	8.10 (2.49)	7.92 (2.55)	8.13 (2.42)
SWL	23.00 (7.32)	20.23 (7.22)	20.00 (7.47)	21.62 (7.73)	22.79 (7.16)	21.57 (7.50)	21.40 (6.75)	20.81 (6.86)	21.12 (7.69)	23.56 (6.95)	21.12 (7.28)	22.45 (7.25)	21.14 (7.43)

### 3.3 Test for dual-factor model of mental health

**Figure 1:**
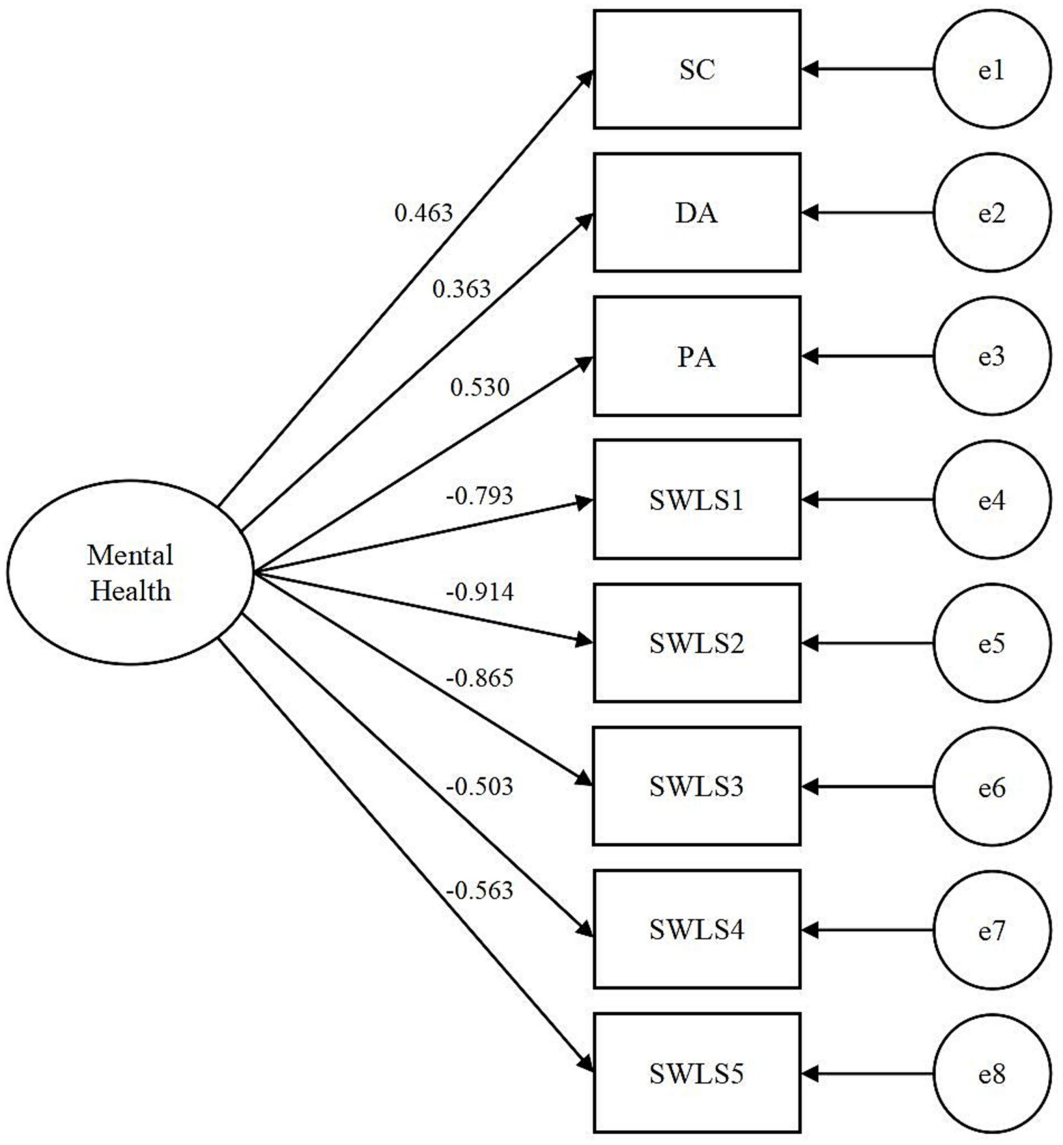
Single-factor model of mental health (Model 1). SC, somatic complaints; DA, depressive affect; PA, positive affect; SWLS1-5, five items of *SWLS.*

**Figure 2:**
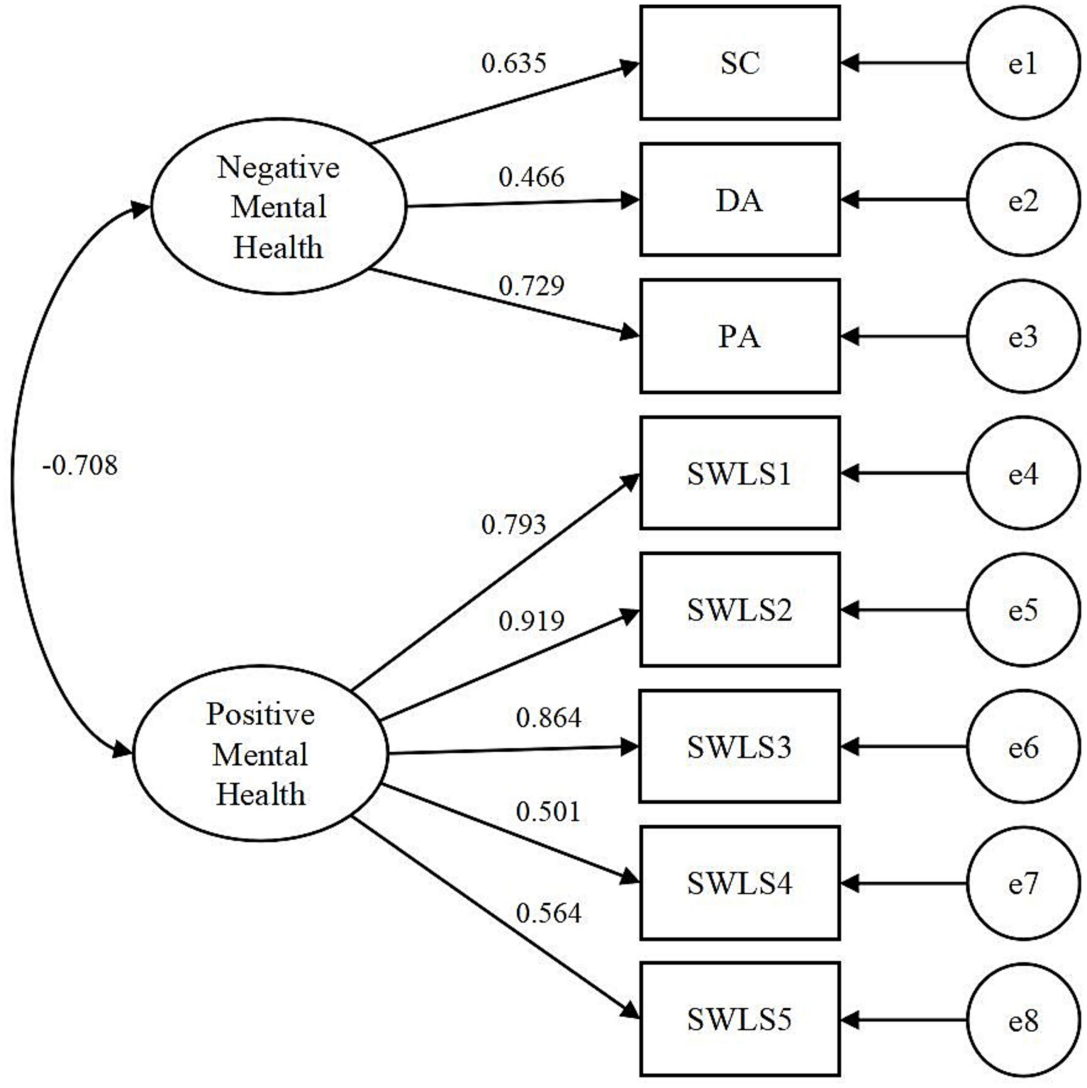
Dual-factor model of mental health (Model 2). SC, Somatic complaints; DA, depressive affect; PA, positive affect; SWLS1-5, five items of *SWLS*.

To evaluate the rationality of the dual-factor model and validate its superior measurement of mental health compared to the traditional model, this study tested two models, as illustrated in Figures 1, 2. Model 1 represented the single-factor model of mental health, in which a latent variable-mental health-represented overall mental health. Negative aspects of three dimensions of *CES-D* and positive aspects of five items of *SWLS* were loaded onto this latent variable. Model 2 represented the dual-factor model of mental health, encompassing two latent variables: negative mental health and positive mental health. In this model, negative aspects of three dimensions of *CES-D* were loaded onto negative mental health, while positive aspects of five items of *SWLS* were loaded onto positive one.

To verify the rationality of the dual-factor model of mental health, this study conducted a comparison between Model 1 and Model 2, using CFA in M*plus* 8.3. The overall model fit was determined by various goodness-of-fit statistics, as well as its chi-square value. The outcomes are presented in Table 2. The results demonstrate that the fit indices of the dual-factor model of mental health surpass those of the single-factor model.

**Table 2:** Model fit of different mental health models.

Fit indices	χ^2^/*df*	*CFI*	*TLI*	*RMSEA*	*SRMR*
Model 1	12.992	0.966	0.947	0.084	0.044
Model 2	4.718	0.990	0.983	0.047	0.020

χ^2^, chi-square; df, degrees of freedom; CFI, comparative fit index; TLI, Tucker-Lewis index; RMSEA, root mean square error of approximation; SRMR, standardized root mean square residual.

### 3.4 Identification of mental health profiles

The results of LPA are displayed in Table 3. Starting with a one-class model (C1), we gradually increased the number of potential classes, and fitted these potential class models separately. Notably, the values of AIC, BIC, and aBIC consistently decreased as the number of classes increased. However, based on the criterion that the smallest class should contain at least 5% of the total sample, the four-profile and five-profile solutions were rejected since the smallest class only accounted for 3.4 and 3.6% of the total sample, respectively. The entropy of the three-profile solution was higher than that of all other models. Therefore, the values of AIC, BIC, aBIC, entropy, and the smallest class sample size suggest that the three-profile solution is likely the most appropriate for our data.

**Table 3:** Fit statistics, classification indices, and class sizes for each model.

Class	AIC	BIC	aBIC	Entropy	LMR (*p*)	BLRT (*p*)	Class proportions
1	19105.236	19148.658	19123.243	-	-	-	-
2	17551.203	17621.764	17580.464	0.825	<0.001	<0.001	0.269/0.731
3	17144.782	17242.482	17185.298	0.836	<0.001	<0.001	0.315/0.613/0.072
4	17009.324	17134.162	17061.094	0.826	0.008	<0.001	0.543/0.114/0.309/0.034
5	16862.440	17014.417	16925.465	0.777	0.043	<0.001	0.523/0.169/0.124/0.148/0.036

AIC, Akaike information criterion; BIC, Bayesian information criterion; aBIC, adjusted Bayesian information criterion; LMR, Lo–Mendell–Rubin adjusted LRT test; BLRT, bootstrapped likelihood ratio test; p, probability estimate.

As shown in Table 4, the average membership probabilities for each latent class consistently surpass 0.80, indicating a high degree of classification accuracy.

**Table 4:** Average membership probabilities (columns) for each latent class (row).

Class	C1	C2	C3
C1	0.888	0.085	0.027
C2	0.052	0.948	0.000
C3	0.087	0.000	0.913

**Figure 3:**
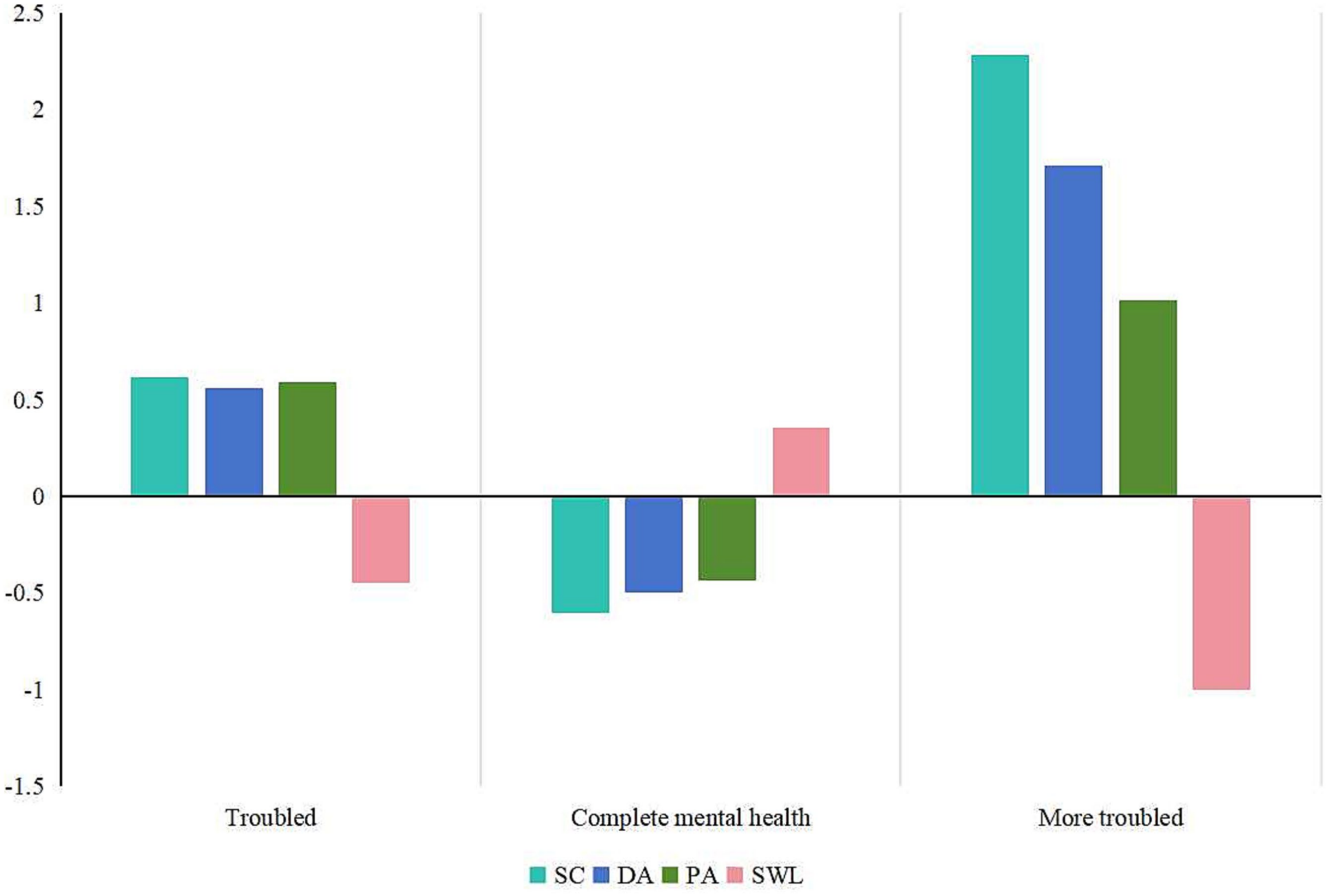
Latent profiles of mental health based on dual-factor model. SC, somatic complaints; DA, depressive affect; PA, positive affect; SWL, satisfaction with life.

Figure 3 presents the estimated mean plots for the three-profile solution in our data. Based on the pattern of mean scores across SC, DA, PA, and SWLS, the three profiles can be labeled as follows: (1) Troubled. Members of this group exhibit high levels of negative mental symptoms and low levels of positive mental experiences. This category comprises 530 individuals, accounting for approximately 31.51% of the total population. (2) Complete mental health. Members of this group show low levels of negative mental symptoms and high levels of positive mental experiences. This category includes 1,031 individuals, making up about 61.30% of the total population. (3) More troubled. Members of this group demonstrate higher levels of negative mental symptoms and lower levels of positive mental experiences. This category consists of 121 individuals, representing around 7.19% of the total population.

### 3.5 Predictive role of emotional regulation

Results of multinomial logistic regression of emotional regulation on profiles are presented in Table 5. We focus on two outcome comparisons:complete mental health vs. troubled and more troubled vs. troubled, with cognitive reappraisal and expressive suppression as predictors. For the complete mental health vs. Troubled comparison: cognitive reappraisal (*b* = 0.092, *SE* = 0.013, χ^2^ = 7.329, *p* < 0.001, *OR* = 1.096, 95% *C. I.* [1.070, 1.124]) is a significant positive predictor. A one-unit increase in it raises the odds of being moderately mentally healthy (vs. symptomatic) by 9.6%. Expressive suppression (*b* = −0.147, *SE* = 0.015, χ^2^ = −9.601, *p* < 0.001, *OR* = 0.863, 95% *C. I.* [0.838, 0.890]) is a significant negative predictor. A one-unit increase in it reduces the odds by 13.7%.

**Table 5:** Multinomial logistic regression of emotional regulation on profiles.

Model	Predictor	*b*	*SE*	χ^2^	*p*	*OR*	*LLCI*	*ULCI*
Complete mental health vs. troubled	Cognitive reappraisal	0.092	0.013	7.329	<0.001	1.096	1.070	1.124
	Expressive suppression	−0.147	0.015	−9.601	<0.001	0.863	0.838	0.890
More troubled vs. troubled	Cognitive reappraisal	−0.106	0.024	−4.415	<0.001	0.899	0.858	0.943
	Expressive suppression	0.088	0.022	3.964	<0.001	1.092	1.045	1.140

b, coefficient of regression; SE, standard error; χ^2^, chi-square; p, probability estimate; OR, odd ratio; LLCI, lower limit confidence interval; ULCI, upper limit confidence interval.

For the more troubled vs. troubled comparison: cognitive reappraisal (*b* = −0.106, *SE* = 0.024, χ^2^ = −4.415, *p* < 0.001, *OR* = 0.899, 95% *C. I.* [0.858, 0.943]) is a significant negative predictor. A one-unit increase lowers the odds of being troubled (vs. symptomatic) by 10.1%. Expressive suppression (*b* = 0.088, *SE* = 0.022, χ^2^ = 3.964, *p* < 0.001, *OR* = 1.092, 95% *C. I.* [1.045, 1.140]) is a significant positive predictor. A one-unit increase raises the odds by 9.2%.

In summary, cognitive reappraisal promotes moderate mental health and reduces the likelihood of being troubled, while expressive suppression shows the opposite trend. These findings highlight the distinct roles of these two emotional regulation strategies in mental health outcomes.

## 4 Discussion

This study employed LPA and multinomial logistic regression to examine the latent profiles derived from the DFM of mental health and the impact of emotional regulation on these mental health profiles.

The DFM was essential to this study, as it integrated subjective well-being and depressive symptoms to capture the full spectrum of adolescent mental health and address gaps in traditional models regarding negative functioning, enabled precise identification of associations between emotion regulation strategies by distinguishing well-being from distress, and overcame limitations of cutoff-based categorization via LPA to identify three distinct profiles, reveal cultural variations, and enhance ecological validity for targeted policies in southwestern China.

Three distinct mental health profiles were identified in this study: troubled, compete mental health, and more troubled. This finding of three-profile of mental health aligns with prior research on early and middle adolescents, such as Zhou et al. (2020), who reported three profiles in Chinese early adolescents, and Clark and Malecki (2022), who identified three profiles in US adolescents. In contrast to the four categories proposed in the dual-factor model of mental health, the latent profiles identified in the present study lack the vulnerable and symptomatic but content groups. The absence of a symptomatic but content profile, observed in studies with older adolescents (e.g., Moore et al., 2019; Gregory et al., 2024), may reflect developmental stage differences. As suggested by Kim et al. (2018), mental health complexity increases with age, and symptomatic but content profiles may emerge later in adolescence as individuals develop more nuanced emotional experiences. The absence of the vulnerable profile and the presence of the more troubled profile may probably reflect the characteristics of the sample.

According to the DFM, individuals are empirically classified into distinct mental health groups with significant functional differences. Those with complete mental health demonstrate the most optimal outcomes across academic, social, and physical health domains (Antaramian et al., 2010; Suldo et al., 2011; Kelly et al., 2012). However, the troubled group consistently experiences the most severe deficits, including the poorest academic performance and lowest levels of social support, marking them as the high-risk category requiring comprehensive intervention (Suldo and Shaffer, 2008; Petersen et al., 2020). In this study, the complete mental health group likely represents adolescents with optimal academic performance, strong social support networks, and minimal future mental health risk, serving as a benchmark for resilience-building programs. The troubled profile may experience moderate functional impairment and require targeted support to prevent symptom escalation. Critical distinctions emerge in the more troubled subgroup. This highest-risk cohort likely faces severe academic disruption, diminished social support, and elevated risks of self-harm or persistent mental illness. Their severe symptoms/extremely low well-being pattern signals urgent need for clinical intervention and resource allocation, distinguishing them qualitatively from the troubled group.

The three-profile solution also underscores the utility of LPA in addressing methodological critiques of DFM. Traditional DFM relies on arbitrary cutoff scores to dichotomize well-being and psychopathology, which may oversimplify mental health heterogeneity (Petersen et al., 2020; Magalhães, 2024). In contrast, our data-driven LPA avoids forced categorization, revealing a dominant complete mental health profile (61.30%), which aligns with Magalhães’ observation that complete mental health or its variants are the most prevalent in community samples (Magalhães, 2024). This prevalence highlights the importance of promoting positive mental health alongside reducing symptoms, as most adolescents fall into a profile with low distress and high well-being. Notably, the sum of the more troubled profile (7.19%) and troubled profile (31.51%) is more prevalent than in some Western samples (e.g., Gregory et al., 2024: 8%), potentially reflecting cultural or contextual factors in southwestern China, such as weaker family support, insufficient mental health education, or different help-seeking norms. However, cross-study variability in profile number (3–5 groups) persists, likely due to sample characteristics (e.g., age, culture) and methodological choices (e.g., indicators included, LPA criteria; Moore et al., 2019). Future longitudinal studies should explore how these profiles evolve across adolescence and whether transitions are influenced by contextual factors like school environment or family dynamics.

The multinomial logistic regression results highlight distinct roles of cognitive reappraisal and expressive suppression in shaping mental health profiles, consistent with Gross’s emotion regulation theory (Gross, 1998, 2015). Gross distinguishes between antecedent-focused strategies (e.g., cognitive reappraisal, which modifies emotional responses by reinterpreting stimuli) and response-focused strategies (e.g., expressive suppression, which inhibits emotional expression post-activation).

Cognitive reappraisal, emerging as a protective factor, positively predicted membership in the complete mental health profile (vs. troubled) and negatively predicted the more troubled profile (vs. troubled). This aligns with extensive evidence that cognitive reappraisal enhances psychological well-being by reducing negative affect and increasing positive emotions (Gross and John, 2003; Aldao et al., 2010). For example, Garnefski and Kraaij (2006) found that frequent use of cognitive reappraisal correlates with lower depressive symptoms, as it fosters adaptive coping and school connectedness (Zhao and Zhao, 2015). In contrast, expressive suppression negatively predicted moderate mental health and positively predicted the troubled profile, showing a maladaptive pattern. While some cross-cultural studies suggest expressive suppression may be less harmful in collectivist cultures (Soto et al., 2016; Zhou et al., 2020), our findings align with Western research labeling it as a maladaptive strategy (Gross and John, 2003; Aldao et al., 2010). This discrepancy may reflect developmental specificity. In adolescents, suppressing emotional expression could disrupt social bonding and increase internalizing symptoms, regardless of culture (Liu et al., 2024). Alternatively, the context of middle school may amplify the costs of suppression, as it hinders authentic emotional communication (Dryman and Heimberg, 2018). These findings reinforce the importance of targeting emotional regulation in interventions. Programs teaching cognitive reappraisal skills could strengthen adolescents’ capacity to maintain well-being, while reducing reliance on suppression may mitigate distress.

This study makes two primary theoretical contributions and reveals potential mechanisms underlying the observed associations. Theoretically, it challenges the universality of the traditional four-group dual-factor model (DFM) by identifying three distinct mental health profiles among Chinese adolescents, absent the vulnerable and symptomatic but content subgroups. Methodologically, the LPA approach advances mental health assessment by avoiding arbitrary cutoff scores, offering a more ecologically valid representation of mental health heterogeneity compared to traditional DFM categorization. Regarding mechanisms, the differential associations between emotion regulation strategies and profiles align with Gross’s process model. Cognitive reappraisal’s protective effect likely operates through antecedent-focused modification of emotional appraisals, reducing negative affect while enhancing positive emotional experiences and psychological flexibility. In contrast, expressive suppression’s detrimental impact may stem from its response-focused nature, which amplifies physiological arousal and cognitive load while inhibiting social bonding, and these processes are exacerbated in collectivistic contexts. These mechanisms highlight emotion regulation as a proximal pathway linking cultural contexts to mental health outcomes.

## 5 Strengths and limitations

The study perhaps have some strengths. First, this study contributes to the debate on the structure of the DFM of mental health: via a data-driven LPA, it identified three profiles, deviating from the traditional four-profile DFM as the vulnerable and symptomatic but content profiles were not detected in the sample. Second, the findings hold practical implications for adolescent mental health practice in southwestern China. The high prevalence of the troubled and more troubled profiles highlights an urgent need for targeted interventions, and schools/communities should prioritize early screening using LPA-derived profiles to identify at-risk students, especially those in the severely symptomatic more troubled subgroup.

The study has several limitations. First, the sample was restricted to junior middle school students in southwestern China, limiting generalizability to other regions or age groups. Second, the cross-sectional design precludes causal inferences about how emotional regulation strategies shape mental health profiles over time; longitudinal studies with repeated assessments are needed to clarify directionality. Third, mental health was assessed using only the depressive symptoms and life satisfaction, omitting other critical indicators such as anxiety, social functioning, or positive affect beyond life satisfaction, which may capture additional profile nuances.

## 6 Conclusion

This study explored latent mental health profiles and their association with emotional regulation among Chinese junior middle school students using the dual-factor model. LPA identified three distinct profiles: complete mental health, troubled, and more troubled, differing from the traditional four-profile dual-factor model. Cognitive reappraisal positively predicted complete mental health and negatively predicted more troubled. Expressive suppression negatively predicted complete mental health and positively predicted more troubled. Conclusions highlight cognitive reappraisal as a protective factor for mental health, while expressive suppression correlates with poorer outcomes, emphasizing the need for targeted interventions promoting cognitive reappraisal to enhance adolescent mental health in southwestern China.

## Data Availability

The raw data supporting the conclusions of this article will be made available by the authors, without undue reservation.
